# Anti-bacterial antibody and T cell responses in bronchiectasis are differentially associated with lung colonization and disease

**DOI:** 10.1186/s12931-018-0811-2

**Published:** 2018-05-30

**Authors:** Fathia G. Jaat, Sajidah F. Hasan, Audrey Perry, Sharon Cookson, Santosh Murali, John D. Perry, Clare V. Lanyon, Anthony De Soyza, Stephen M. Todryk

**Affiliations:** 1 Faculty of Health & Life Sciences, Northumbria University, Newcastle upon Tyne, NE1 8ST UK; 2 Department of Microbiology, Freeman Hospital, Newcastle upon Tyne, NE7 7DN UK; 3 Adult Bronchiectasis Service, Freeman Hospital, Newcastle upon Tyne, NE7 7DN UK; 4 Institute of Cellular Medicine, Newcastle University, Newcastle upon Tyne, NE2 4HH UK; 5 Zawia University, Zawia, Libya; 6 College of Pharmacy, University of Kerbala, Kerbala, Iraq

**Keywords:** Bronchiectasis, Antibodies, T cells, Lung function, Exacerbation, COPD

## Abstract

**Background:**

As a way to determine markers of infection or disease informing disease management, and to reveal disease-associated immune mechanisms, this study sought to measure antibody and T cell responses against key lung pathogens and to relate these to patients’ microbial colonization status, exacerbation history and lung function, in Bronchiectasis (BR) and Chronic Obstructive Pulmonary Disease (COPD).

**Methods:**

One hundred nineteen patients with stable BR, 58 with COPD and 28 healthy volunteers were recruited and spirometry was performed. Bacterial lysates were used to measure specific antibody responses by ELISA and T cells by ELIspot. Cytokine secretion by lysate-stimulated T cells was measured by multiplex cytokine assay whilst activation phenotype was measured by flow cytometry.

**Results:**

Typical colonization profiles were observed in BR and COPD, dominated by *P.aeruginosa, H.influenzae, S.pneumoniae* and *M.catarrhalis.* Colonization frequency was greater in BR, showing association with increased antibody responses against *P.aeruginosa* compared to COPD and HV, and with sensitivity of 73% and specificity of 95%. Interferon-gamma T cell responses against *P.aeruginosa* and *S.pneumoniae* were reduced in BR and COPD, whilst reactive T cells in BR had similar markers of homing and senescence compared to healthy volunteers. Exacerbation frequency in BR was associated with increased antibodies against *P. aeruginosa, M.catarrhalis and S.maltophilia*. T cell responses against *H.influenzae* showed positive correlation with FEV_1_% (*r* = 0.201, *p* = 0.033) and negative correlation with Bronchiectasis Severity Index (*r* = − 0.287, *p* = 0.0035).

**Conclusion:**

Our findings suggest a difference in antibody and T cell immunity in BR, with antibody being a marker of exposure and disease in BR for *P.aeruginosa*, *M.catarrhalis* and *H.influenzae,* and T cells a marker of reduced disease for *H.influenzae*.

**Electronic supplementary material:**

The online version of this article (10.1186/s12931-018-0811-2) contains supplementary material, which is available to authorized users.

## Background

The chronic lung diseases of bronchiectasis (BR) and chronic obstructive pulmonary disease (COPD) are both associated with recurrent airway infections. COPD is a major cause of death globally, with numbers of deaths rising [1], and BR is underestimated with incidence rising in the UK by around 6% annually [2]. Whilst they differ in disease causation, established disease in both is mainly characterised by repeated or persistent heavy bacterial colonization of the damaged lower respiratory tract. Such infection is associated with inflammation, mucus production, and reduced ciliary action, which promotes further infection, inflammation and tissue damage, in a vicious cycle [3]. Studies have suggested that infection causes disease exacerbation and diminished lung function, which are often proportional to the bacterial load and to reduced diversity [4, 5]. More recent findings propose more species-rich lung ecologies where alterations in specific bacterial populations, dysbiosis, is at the heart of clinical disease [6, 7]. Pathogenic bacteria, as determined clinically by microbiological culture of expectorated sputum, are dominated by organisms specific to these diseases including *Pseudomonas aeruginosa, Haemophilus influenzae, Streptococcus pneumoniae* and *Moraxella catarrhalis* [8]. Recent studies using DNA-sequencing technology reveal more detailed bacterial ecosystems in the lungs of diseased patients, but with culture approaches mainly corroborated [9, 10]. *P.aeruginosa* is considered the major cause of morbidity (increased exacerbations and reduced lung function) and mortality in BR [11], particularly during chronic infection and mucoid characteristics of the bacterium [12], which may allow evasion of host immunity. Non-typeable strains of *Haemophilus influenzae* (NTHi) are frequently found in BR [13] and are not targeted by current vaccines. Both pathogens are also common in COPD albeit with a reduced frequency of Pseudomonas infections as compared to BR [14]. Furthermore, less frequent suppurative infection and sputum production in COPD results in lower detection of pathogenic microbes, implying fewer infections than BR. Failure to produce sputum for microbiology, particularly in younger BR patients and in many COPD patients, as well as intermittent negative cultures, means that immune biomarkers of disease may provide a useful adjunct for directing clinical management.

Knowledge of immunity in BR is limited, but studies suggest immune system genes that are involved in presentation of antigens to CD4^+^ T cells, such as HLA-DR1 and DQ5, play a role [15, 16]. Notably, a role for adaptive immune responses (specific antibodies and T cells) in protection against *P.aeruginosa and H.influenzae,* has been demonstrated in human vaccine trials in cystic fibrosis-related bronchiectasis [17, 18] and in mouse vaccination models [19, 20]. Furthermore, the above-mentioned lung pathogens appear in individuals with defined immunodeficiencies [21], underlining the role of antibodies and phagocytes in protection. Whilst healthy individuals are exposed to the same pathogenic organisms as diseased individuals, healthy lungs typically have low levels of bacterial species, reflecting the naso-pharynx [22]. Immune responses against pathogenic microbes do not cause overt immunopathology in healthy individuals, but may contribute to disease in colonized patients due to continuous immune stimulation by the localised high antigen doses, particularly through excessive Th17 responses that promote neutrophil infiltration [23]. Together with inflammatory cytokines, neutrophils are abundant in the sputum of BR patients, and decline after antibiotic treatment [24]. It is possible that dysfunction of both innate and adaptive immunity contribute directly or indirectly to disease in both BR and COPD. The aim of this study was to characterise antibody and T cell responses against key lung microbes in disease-stable patients with BR and COPD, characterised by the Bronchiectasis Severity Index (BSI) and GOLD guidelines, respectively, in comparison to controls (healthy volunteers), and to relate the immune responses to culture-based bacterial colonization, lung function and frequency of exacerbation.

## Methods

### Study participants and samples

Ethical approval for the project was granted by the local NHS Research Ethics Committee, the NRES Committee North East – County Durham & Tees Valley (ref 12/NE/0248). Adult patients with (non-CF) BR, COPD and healthy volunteer (HV) controls, were recruited at the Freeman Hospital, Newcastle upon Tyne. Female to male ration was about 1.5:1. BR is routinely confirmed by high-resolution computed tomography (HCRT), and COPD according to prevailing GOLD guidelines (BTS and NICE 2010, [25]). Diverse aetiologies of BR were included in the study, with the exception of known immunodeficiency.

Patients were clinically stable at the time of assessment. They underwent spirometry to determine forced expiratory volume in 1 s (FEV_1_), and Forced Vital Capacity (FVC), from which FEV1% predicted, FEV1/FVC ratio and FVC % predicted were obtained. The bronchiectasis severity index (BSI) score, as previously validated [26], was assessed. Patients were divided into 2 groups: either those with one severe exacerbation requiring hospitalisation or those with 3 or more exacerbations per year, compared to those not requiring hospitalisation and having less than 3 exacerbations per year. The exacerbations were determined for the preceding 12 months. Colonization history of patients was also available going back at least 4 years. Patients were categorised by pathogen status based on positive sputum cultures. ‘Chronic colonization’ was defined here as 2 positive sputum cultures at least 3 months apart in 12 months. ‘Chronic currently’ was defined as a positive sputum culture at time of blood sampling (for immune responses), and more than 2 positive sputum cultures in 12 months. ‘Previously chronic’ was defined as more than 2 positive sputum cultures in 12 months > 2 years ago. ‘Occasional’ infection was ≥ 1 positive sputum culture per year. ‘No colonization’ was sputum culture negative over at last 3 years (Table 3).

### Sample processing and bacterial culture

Heparinized venous blood samples from patients and healthy controls were processed to give plasma for ELISA and peripheral blood mononuclear cells (PBMC) for T cell assays (detailed in Additional file 1). Sputum samples were cultured in the Microbiology Department, Freeman Hospital, according to national standards.

### Enzyme-linked immunosorbent assay (ELISA) for serum antibody measurement

An indirect enzyme-linked immunosorbent assay (ELISA) method was used: to determine optimal dilutions for the coating of microbe-derived antigens, for initial serum screening, and to undertake titration for total IgG (all subclasses combined) to give an end-point titre (e.g. 1 in 1000), and for the measurement of individual Ig subclasses (given as absorbance for 1 in 25 dliution), as described previously [27](see Additional file 1).

### T cell responses to microbial antigens

ELIspot as previously optimised and described [28] (see Additional file 1) was used to measure T cell responses against a range of stimuli including bacterial lysates plus selected peptide epitopes where available. For cell activation and surface staining of cells, PBMC were thawed and activated with stimuli for 20 h as detailed in the supplement. Intracellular staining was performed to determine the number and phenotype of IFNγ-producing or activated CD69^+^ T cells following stimulation.

### Measurement of cytokines in culture supernatants using multiplex ELISA

Cytokines in supernatants from stimulated PBMCs were measured using the Mesoscale Scale Discovery (MSD) multiplex cytokine Kit (Meso Scale Diagnostic, LLC, Gaithersburg, USA). Multiplex kit – pro-inflammatory panel 1 (for IL-1β, IL-2, IL-4, IL-6, IL-8, IL-10, IL-12p70, TNFα) and cytokine panel 2 (IL-17A, IL-5) were used. See Additional file 1.

### Statistics

The immunological data was tested for normal distribution using the Shapiro-Wilk test. For normally distributed data t-test and Pearson correlation was used whilst for not normally distributed data the Mann-Whitney U test and Spearman’s correlation was used. A priori calculations based on our previous data suggested that sufficient numbers were included to detect a modest effect with 0.9 power to a significance level of 0.05. SPSS v.15 and Graph Pad Prism were used for analysis. The cut-off value for statistical significance was *p* < 0.05.

## Results

### Clinical data

This study examined antigen-specific immune responses in 119 BR and 58 COPD patients, and in 28 HV (Table 1), against lung pathogens that are commonly isolated from these patient groups. The patient groups showed typical clinical features, similar to previously-published reports e.g. average FEV_1_% predicted, which was 68 for BR, 49 for COPD and 113 for HV. FVC % predicted and FEV1/FVC ratio were also reduced in the patient groups compared to HV. The BR group was predominantly of a post-infection aetiology, whilst COPD was smoking-related, and all patients with BR and COPD were in a clinically stable state with no current exacerbation. At the time of taking the blood samples, the BR group had a higher proportion of patients producing sputum (93%) that could be microbiologically tested than COPD (66%), and BR patients showed greater overall populations infected with the main bacterial species (Table 2), many with multiple species. *H.influenzae* was the most commonly identified species in both BR and COPD, followed by *P.aeruginosa*, *S.pneumoniae* and *M.catarrhalis* in BR, and *M.catarrhalis, S.pneumoniae* and *P.aeruginosa* in COPD.

**Table 1:** Demographics of the subjects included in this study

Characteristics	BR *n* = 119	COPD *n* = 58	HV *n* = 28
Sex (no.) Male/female)	45/74	Not av.	Not av.
Age (y)	65 ± 1.08	69 ± 1.23	54 ± 3.01
Exacerbations (per year)	4 ± 0.29	3 ± 0.38	Not app.
Smoking history (pack years)	8 ± 1.33	47 ± 4.08	Not av.
FEV_1_ (% predicted)	68 ± 2.68	49 ± 2.70	113 ± 2.83
FVC (% predicted)	82 ± 2.48	77 ± 2.40	118 ± 2.7
FEV_1_/FVC ratio	66 ± 1.52	50 ± 2.17	83 ± 1.75

Values are presented as means ± SEM, exacerbations represents the number per year. FEV_1_ represent forced expiratory volume in the first second and FVC, forced vital capacity. Not av., indicates the data are not available, whereas Not app., means that the category was not applicable

**Table 2:** Microorganisms isolated from the sputum of patients with BR and COPD

Microorganism Identified	Bronchiectasis patients (%)	Chronic obstructive pulmonary disease patients (%)
NT*. H.influenzae*	63.8	31
*P. aeruginosa*	56.3	12.0
*S. pneumoniae*	44.5	17.2
*M. catarrhalis*	37.8	24.1
*A. fumigatus*	22.6	3.4
*S. maltophilia*	15.9	3.4
*Candida.* sp	14.4	6.9
*S aureus*	30.3	1.7
*E.coli*	20.1	5.2
No pathogen isolated	2.5	17.2
No sputum produced	6.8	43.7

BR patients n = 119; COPD patients n = 58

*NT* = non-typeable

### Antibody responses

**Fig. 1:**
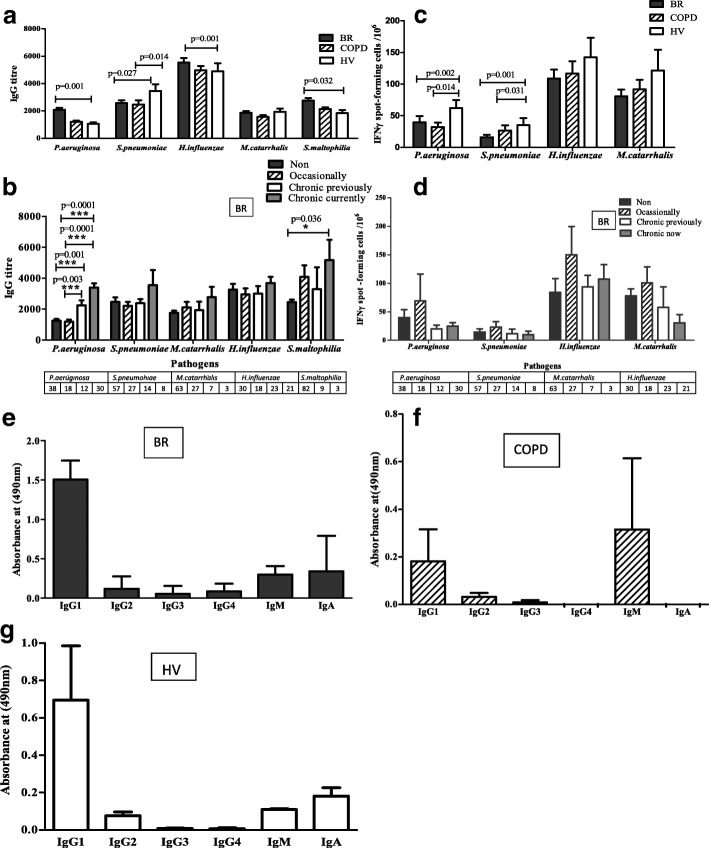
Anti-bacterial antibody and T cells responses. Antibody levels against bacterial antigens were measured by ELISA: (**a**) IgG titres (1/value) against bacteria in BR, COPD and HV groups; (**b**) Antibody responses against bacteria were compared based on bacterial colonization. T cell responses against bacterial antigens were measured by IFNγ ELIspot: (**c**) IFNγ spot-forming cells per 10^6^ PBMC against bacteria in BR, COPD and HV groups; (**d**) IFNγ spot-forming cells per 10^6^ PBMC against bacteria were compared based on bacterial colonization. Anti-pseudomonas Ig subclasses and IgG isotypes in BR (**e**), COPD (**f**) and HV (**g**) subgroups. Mean value + SEM are given. *p < 0.05, ***p < 0.001. Mann-Whitney tests were performed

The first experiments involved ELISA to determine antibody levels (total specific IgG), by end-point titre, against the main bacterial species in BR, COPD and HV. BR showed significantly higher levels of antibody against *P.aeruginosa*, *H.influenzae* and *S.maltophilia* compared to HV (Fig. 1a). COPD failed to show significant increase from HV for any of the bacteria. HV only showed significantly higher antibody responses than BR and COPD against *S.pneumoniae*.

The next aim was to relate specific IgG antibody levels against bacteria, to bacterial colonization status and history. Because of the relatively low infection rates in COPD, the numbers were insufficient to determine significance, and so we focussed on BR. BR patient pathogen status was analysed and condensed into a categorization shown in Table 3, indicating ‘current chronic’, ‘previous chronic’, ‘occasional’, or ‘no colonization’. Whilst there was an overall trend for antibody titre to increase based on colonization (Fig. 1b), this was only significant for *P.aeruginosa* and *S.maltophilia*. We further studied immunoglobulins by class and isotype across the disease groups and found in general higher levels of IgG1 and IgA in BR than COPD (Fig. 1e-g).

**Table 3:** Classification of patients based on sputum microbiological results

0	No pathogen isolated (NPI)	
1	Occasional	≥ 1 isolation in a year
		Chronic colonization is defined by the isolation of bacteria in sputum culture on 2 or more occasions, 3 months apart, within 1 year
2	Chronic previously	Chronic in preceding 5 years but not last 2 years
3	Chronic currently	Current, and chronic in last 2 years prior to recruitment

### T cell responses

T cell responses, in the form of IFNγ spot-forming cells per 10^6^ PBMC, were measured against the bacterial antigens as for the antibodies. In contrast to the antibody responses, there was an overall trend towards the BR and COPD groups having lower T cell responses than the HV group (Fig. 1c). BR and COPD both had significantly lower responses than HV against *P.aeruginosa* and *S.pneumoniae*. As with the antibody responses, the relatively low infection rates in COPD meant that there were insufficient numbers of infected patients to determine significance and so BR was focussed upon for relating to colonization (Fig. 1d). Whilst there was an overall trend for T cell responses against all antigens to associate with an occasional number of exposures, as opposed to none or chronic, this was not significant.

### Exacerbation and lung function

**Fig. 2:**
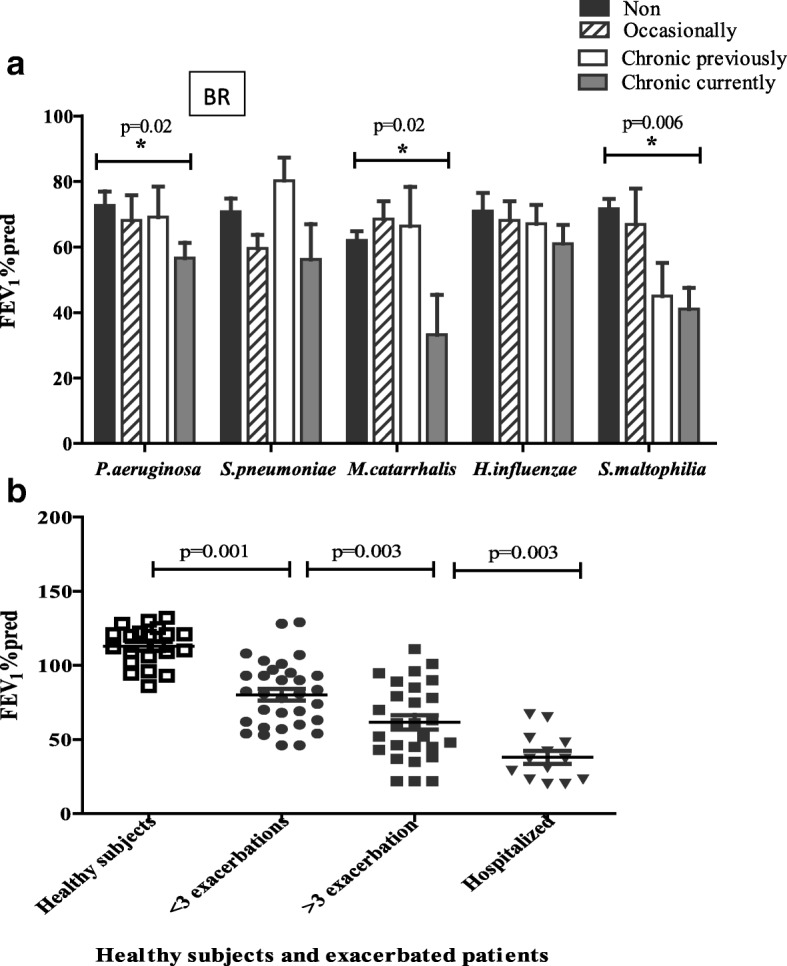
Lung function and exacerbation in BR. FEV1% predicted is a measure of lung function. (**a**) FEV1% predicted in BR patients grouped based on their bacterial colonization. (**b**) FEV1% predicted in BR patients based on exacerbation in the last year, and in HV – healthy subjects. Mean values + SEM are given. Mann-Whitney tests were performed

The contribution of bacterial colonisation, as per Table 3, to lung function as measured by FEV_1_% predicted, was examined in the BR cohort. Overall there was a downward trend in lung function as bacterial colonisation became more frequent and with current positive cultures (Fig. 2a), with *P.aeruginosa, M.catarrhalis* and *S.maltophilia* all showing a significant reduction. The numbers with *M.catarrhalis* and *S.maltophilia* were low (*n* = 10 and 9, respectively) with 52 and 75% of these, respectively, being co-colonised with *P.aeruginosa*.

**Fig. 3:**
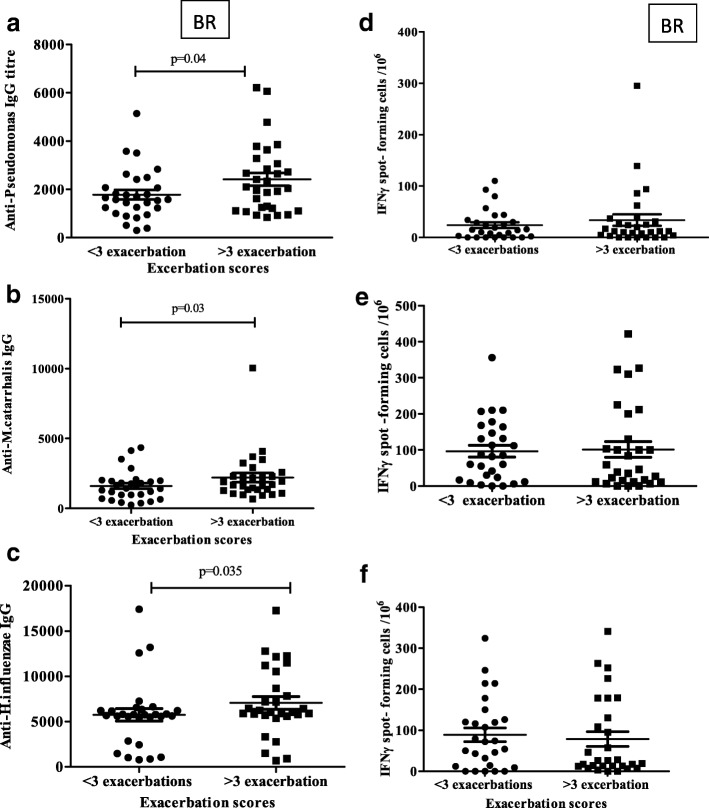
Antibody and T cell responses in BR groups of different recent exacerbation history. Antibody levels against bacterial antigens were measured by ELISA to give IgG titres (1/value) in BR groups with < 3 compared to > 3 exacerbations in the last 12 months against bacteria. T cell responses were measured by IFNγ ELIspot and expressed as spot-forming cells per 10^6^ PBMC. (**a**, **d**) *P.aeruginosa* (**b**, **e**) *M. catarrhalis* (**c**, **f**) *H.influenzae*. Mean values + SEM are given. Mann-Whitney tests were performed

Since exacerbation of disease is a key event in need of urgent clinical management, and makes up part of the validated BSI scoring, exacerbation history of the BR patients was used to determine whether there were any informative associations with immunological responses. The patients were compared based on them having less than 3 exacerbations, greater than or equal to 3, and those that were hospitalised within the last year. This breakdown of the patients was first validated by examining the FEV_1_% predicted within the groups compared to HV (Fig. 2b). A significant decrease in lung function was seen moving from HV, through < 3 exacerbations, ≥ 3 exacerbations, to hospitalised. Since the hospitalised group comprise a variable causality (and not necessarily greater disease) and was relatively small in number, this was omitted from further analysis. Small but statistically significant increases in IgG titres against *P.aeruginosa, H.influenzae* and *M.catarrhalis* were seen between < 3 and ≥ 3 exacerbations (Fig. 3a-c). For T cell responses no significant differences were seen between the 2 groups for any bacterium (Fig. 3d-f).

### T cell phenotypes

**Fig. 4:**
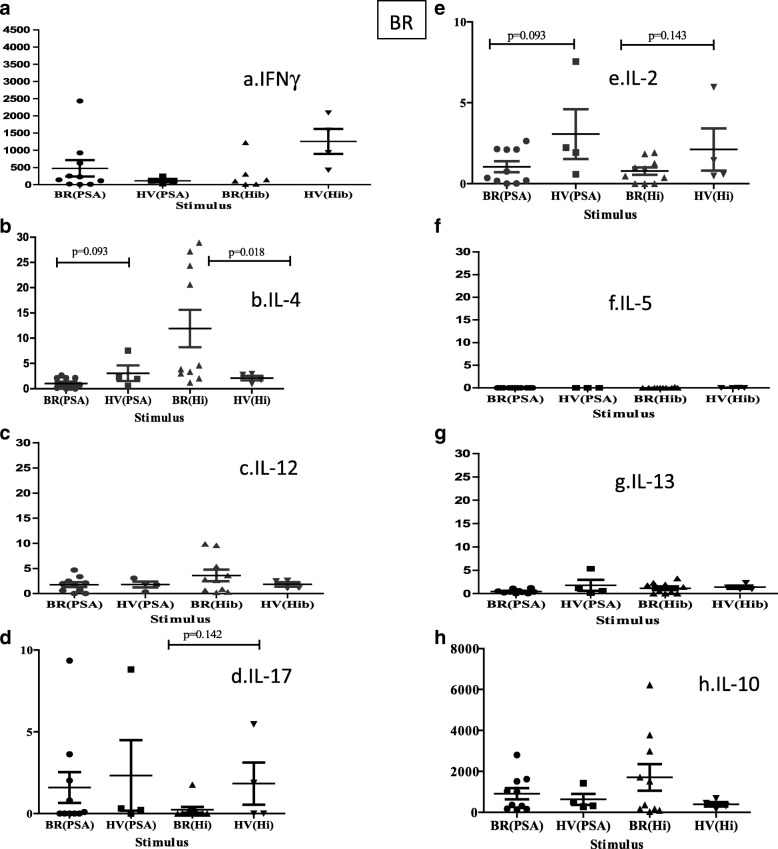
Cytokine responses in a subset of BR patients and HV. PBMC from BR and HV were stimulated with bacterial antigens *P.aeruginosa* (PSA) and *H.influenzae* (Hi), and supernatants were tested for the following cytokines: (**a**) IL-2, (**b**) IL-4, (**c**) IL-12, (**d**) IL-17, (**e**) IL-2, (**f**) IL-5, (**g**) IL-13, (**h**) IL-10. Mean values + SEM are given in pg/ml. Mann-Whitney tests were performed

Since T cell responses of a single Th type (Th1/IFNγ) may not be the only response against the lung bacteria, antigen-specific T cell cytokine responses were also measured in the supernatants of antigen-stimulated PBMC by multiplex cytokine analysis (Fig. 4a-h). A sub-group of BR patients who were good responders to the antigens, determined by both IFNγ ELIspot and by CD69 expression following antigen-stimulation, were selected for stimulation, as well as similarly good HV responders. For IFNγ, IL-2 and IL-17 responses the BR patients showed a trend, though not significant, towards reduced levels for both *P.aeruginosa* and *H.influenzae* compared to HV. For IL-4, BR patients showed marginally reduced responses for *P.aeruginosa* but significantly increased responses to *H.influenzae* compared to HV, although the responses showed considerable variability. Finally, IL-10 responses showed a trend for increased response in BR over HV for both antigens, but again with much variability. All other cytokine responses tested were equivocal.

**Fig. 5:**
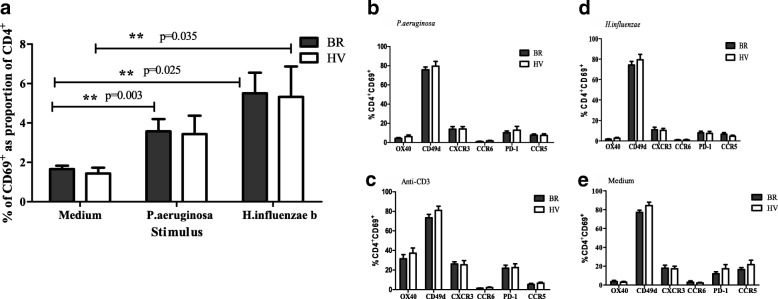
Flow cytometry responses on a subset of BR patients and HV. Flow cytometry was performed on PBMC from BR and HV stimulated with bacterial antigens. (**a**) CD69 activation marker following stimulation with medium only, *P.aeruginosa* or *H.influenzae*. The % of CD4+ CD69+ activated T cells with phenotypic markers following stimulation with (**b**) *P.aeruginosa*, (**c**) anti-CD3 monoclonal antibody, (**d**) *H.influenzae* and (**e**) medium only. Mean % values + SEM are given. Mann-Whitney tests were performed

Flow cytometry analysis was used to further characterise antigen-responding T cells in the same subgroups of BR and HV. Similar proportions of activated CD69^+^ CD4^+^ T cells were seen in BR and HV following stimulation with *P.aeruginosa* and *H.influenzae*, all being significantly higher than the unstimulated (medium) control (Fig. 5a). Co-staining of the activated CD4^+^ T cells with CD69 and for IFNγ intracellularly showed a concordance of the two forms of activation but showed that there were more CD69^+^ cells than IFNγ^+^ cells (Additional file 1). Staining of the CD69^+^ stimulated cells for other potentially important markers (see Additional file 1: Figure S1), showed significant levels of staining (above isotype controls) on CD4^+^ CD69^+^ T cells (Fig. 5b-e), but no significant difference between BR patient and HV cells.

**Fig. 6:**
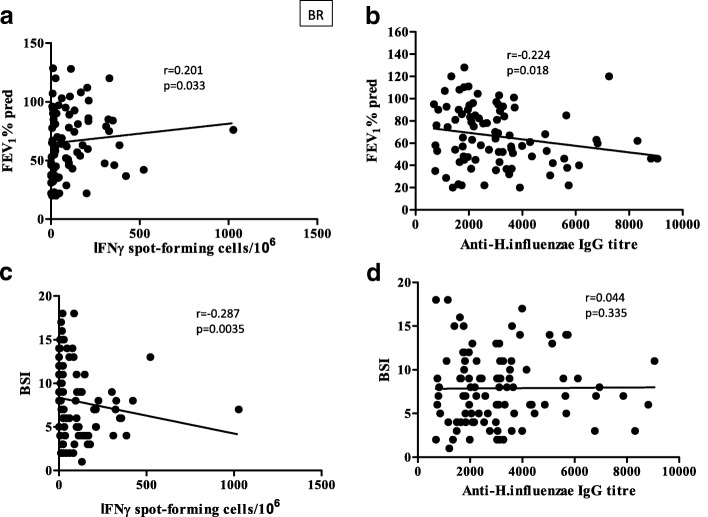
Lung function and BSI related to immune responses against *H.influenzae* in BR. (**a**) T cell responses against *H.influenzae* measured by IFNγ ELIspot and expressed as spot-forming cells per 10^6^ PBMC plotted against FEV1% predicted. (**b**) T cells responses against *H.influenzae* plotted against BSI. (**c**) Antibody levels against *H.influenzae* were measured by ELISA to give IgG titres (1/value) in BR plotted against FEV1% predicted. (**d**) Antibody responses against *H.influenzae* plotted against BSI. Spearman correlation was performed

When the relationship between T cell responses (IFNγ ELIspot) and lung function (FEV_1_% predicted) was examined, a significant positive correlation (Fig. 6a), albeit weak (*r* = 0.201; *p* = 0.033), was found only with T cell response against *H.influenzae*. With removal of the outlier, significance was still retained (*r* = 0.186, *p* = 0.049). Furthermore, a stronger (negative) correlation was observed between BSI and anti-*H.influenzae* T cell responses (Fig. 6c) (*r* = − 0.287; *p* = 0.003), and again significance remained upon removal of an outlier (*r* = − 0.265, *p* = 0.0035). Conversely, a moderate negative correlation was seen (Fig. 6b) with antibodies against *H.influenzae* (*r* = − 0.224; *p* = 0.018), but none with BSI (Fig. 6d). This suggests that T cell responses are associated with improved lung function and less severe disease, whereas higher levels of *H. influenzae* antibodies are associated with poorer lung function. Overall, no relationships were evident between the magnitudes of antibody and T cell responses against any of the bacteria examined. Furthermore, T cell and antibody responses against bacteria showed no relationships with one another (data not shown).

## Discussion

This study began by comparing immune responses against common lung pathogens in BR, COPD and HV. The clinical categorisation of the patients followed standard processes and was in keeping with other studies in the field, as were the microbiology results obtained. One expectation was that the degree of exposure to the microbes will be proportional to the magnitude of immune response measured. This was broadly the case for antibody responses, which were higher in BR than COPD and HV, particularly against *P.aeruginosa*, *H.influenzae* and *S.maltophilia*, reflecting rates of positive sputum cultures in BR and COPD. Measurement of isotype components of the antibody responses against *P.aeruginosa* showed a high IgG1 component in BR and HV, compared to COPD which had a higher IgM. This may suggest that COPD has reduced isotype switching, which is usually controlled by cognate T cell responses, through CD40:CD40L interaction and through cytokines. Reduced or altered antibody responses as we have seen here could be due to increased regulatory T cells, as have been demonstrated in COPD, which may depress protective immunity [29].

Having found specific antibody responses to be increased in BR, the question was whether these responses showed a direct dynamic relationship with colonization levels. Sufficient numbers for this analysis were only available in the BR group. Whilst there was a trend for increasing antibodies with colonisation for each individual pathogen, only *P.aeurginosa* and *S.maltophilia* showed significance. We categorised patients based on their exacerbation frequency (< 3, ≥ 3) which were validated by showing reducing lung function. Although significant, only modest increases in antibody against *P.aeruginosa, M.catarrhalis* and *H.influenzae* were found in BR with ≥ 3 exacerbations compared to < 3. Antibody response only against *H.influenzae* showed a negative correlation with FEV_1_% predicted, suggesting it to be a marker of disease and exposure.

The measurement of T cell responses against lung pathogens may be useful for the diagnosis of latent infection, as is the case of the Quantiferon test for *Mycobacterium tuberculosis* (Mtb). In this study T cell responses showed an overall tendency for reduction in BR and COPD compared to HV, associated with colonisation status, with responses to *P.aeruginosa* and *S.pneumoniae* being significantly reduced. This suggests that increased infection and exposure may exhaust the T cell response. Within the BR group T cell responses showed a trend for being highest in the group that had occasional infections, for all pathogens tested. The highest T cell responses were found for *H.influenzae* and *M.catarrhalis* which coincides with them having intracellular phases that require T cells for efficient immune protection or eradication. T cell responses did not show any associations with exacerbation level. However, increased IFNγ ELIspot T cell responses against *H.influenzae* showed significant positive association, albeit weak, with lung function (FEV_1_%) and negative association with BSI, which may suggest that T cells are protective against disease, in contrast to antibody responses which showed a negative correlation with FEV_1_%, and may simply be associated with more infection. The next aim was to investigate further the nature of the T cells reactive against the two major pathogens, *P.aeruginosa* and *H.influenzae,* in a sub-group of BR patients who were good responders to the antigens and in comparison to good-responding HV. There was a tendency for IFNγ, IL-2 and IL-17 to be reduced in BR patients compared to HV, suggesting greater antigen exposure, where memory T cells producing IL-2 convert to T cells secreting effector cytokines. Conversely, there was a tendency for IL-10 to be increased in BR for both antigens suggesting their conversion to a regulatory (Tr1) phenotype due to high and sustained antigen exposure at the mucosal surface. IL-4 responses showed a significant increase in BR against *H.influenzae*, similar to published work on COPD [30], but a tendency for the opposite for *P.aeruginosa*. This suggests a discrepancy in immune responses between BR and HV, and against the two pathogens, reflecting the fact that T cell response against *H.influenzae* was protective against disease. When pathogen-reactive T cells, based on CD69 and CD4 staining, were examined for further key phenotypic markers no differences were found between BR and HV. All reactive cells had high levels of CD49d, a lung homing receptor, but low levels of inflammatory homing receptors and the marker of senescence PD-1.

The measurement of antibodies and T cells specific for *P.aeruginosa* and *H.influenzae* in patients with BR [31, 32] and COPD [30, 33] has previously revealed increased antibody responses associated with repeated infection, but decreased T cell responses, despite CD4^+^ T cell presence and oligoclonal TcR T cell expansion in the lungs [34, 35], suggesting immune dysregulation such as T cell exhaustion. Thus, while immune responses may be protective, or a marker of infection by microbes, their dysregulation may be detrimental to the patient due to reduced protection from infection or through immunopathology as suggested in cystic fibrosis [36]. Studying responses in disease states is important as this may reveal mechanisms of disease that are direct (via immunopathology) or indirect (via anti-microbial effects) that may provide therapeutic targets. Furthermore, studies of such blood-based immunodiagnostics may be useful for diagnosis and stratification of patients, and their responses to treatment [31], when microbiology or genomic analysis is not possible or reliable (young BR patients, no sputum, difficult to culture microbes, false negative). Baseline immunity related to contemporaneous microbiota may particularly be a useful way to identify a frequent exacerbator phenotype. With regard to an antibody marker of current colonization with *P.aeruginosa*, this data showed 92% specificity (ability to show true negatives) and 73% sensitivity (ability to show true positives) based on the HV mean + 2 sd. This is similar to previous findings [31].

The strengths of the study were the extensive nature of the immunological investigations carried out on patients, particularly those with BR, who were well characterised clinically and microbiologically. One weakness is that numbers of COPD patients producing sputum, and thus with positive cultures, was too low to allow a sufficiently powered analysis to be undertaken for COPD and so the study focussed on BR after the initial observations (Fig.1). Furthermore, it would have been useful to have longitudinal data of immune responses and microbiology, and this is the subject of a future study. Another weakness is that microbiological culture is not able to determine the complete microbial makeup in a sample if it contains fastidious unculturable bacteria. We are currently addressing this by carrying out genomic analysis of patient sputum samples as well as microbiological culture. Finally, the cytokine secretion data would have benefited from larger numbers, particularly for HV, and again this is the subject of ongoing work.

## Conclusion

In conclusion, exposure to these lung pathogens generates antibody responses of magnitudes that are broadly proportional to the level of exposure and thus disease (exacerbation, reduced lung function), and may be useful markers of disease. T cell responses appear to be reduced in patients with increased infection rates, and are proportional to lung function and BSI for *H.influenzae,* suggesting that they may be protective against such a pathogen that is partially intracellular. The T cell responses in patients differ little in phenotype from HV, apart from possible subtle cytokine differences that are currently being examined further. The interaction between T cells and antibody-producing B cells, and how the two arms of the adaptive immune response interact and influence innate immunity, and ultimately impact on bacterial infection and disease, is likely to be complex and multifactorial. The data in this study suggests the use of antibodies for Pseudomonas-inducing disease diagnosis, whilst T cells may indicate protective immunity against Haemophilus, suggesting a possible benefit of T cell-inducing vaccines.

## Additional file

Additional file 1: Supplement: The significance of anti-bacterial immune responses in Bronchiectasis and Chronic Obstructive Pulmonary Disease. (DOCX 235 kb)

## Abbreviations

BR: Bronchiectasis
COPD: Chronic obstructive pulmonary disease
ELISA: Enzyme linked immunosorbent assay
ELIspot: Enzyme linked immunospot
FEV: Forced expiratory volume
Hi: *Haemophilus influenzae*
HV: Healthy volunteers
PSA: *Pseudomonas aeruginosa*
