# Supplementary material for: Anti-bacterial antibody and T cell responses in bronchiectasis are differentially associated with lung colonization and disease
Source: Respir Res. 2018 May 30;19:106. doi: 10.1186/s12931-018-0811-2 (PMC5977760; doi:10.1186/s12931-018-0811-2)
Supplement: Supplementary file 1 — Supplement: The significance of anti-bacterial immune responses in Bronchiectasis and Chronic Obstructive Pulmonary Disease. (DOCX 235 kb) [file 12931_2018_811_MOESM1_ESM.docx]

**The significance of anti-bacterial immune responses in Bronchiectasis and Chronic Obstructive Pulmonary Disease**

Fathia G Jaat^1,5^, Sajidah F Hasan^1,6^, Audrey Perry^2^, Sharon Cookson^1^, Santosh Murali^1^, John Perry^2,1^, Clare V Lanyon^1^, Anthony De Soyza^3,4*^, Stephen M Todryk^1,4*⮹^

**Supplement section**

Heparinized venous blood samples were taken from patients and healthy controls and were processed by layering onto Lymphoprep (Axis-Shield) solution in a Leukopsep tube (Greiner), and centrifugation at 2100 rpm for 12 minutes. Separated plasma was aliquoted and stored at 4^o^C short term or frozen at -20^o^C for longer term storage before use in ELISA. Resulting peripheral blood mononuclear cells (PBMC) were washed in RPMI 1640 and frozen in 90% Foetal Calf Serum / 10% Dimethylsulphoxide at -150^o^C.

**Bacterial Lysate Production**

A panel of reference bacterial strains were provided by the Microbiology Department, Freeman Hospital, for the production of antigen lysates (Supplement Table S1). These strains were cultured from a single plated colony in 10 ml of broth (depending on the species; see Table S1), and incubated with shaking at 200 rpm overnight at 37^o^C. Then, 1 ml of this starting culture was transferred into broth in a 200 ml flask and incubated with further shaking for approximately 2-3h at 37^o^C until the optical density (OD) of the culture reached 0.8, indicating mid-log growth. The bacteria were pelleted by centrifugation at 4000 rpm for 15 min at 4^o^C and then washed 3 times with 50 ml of phosphate buffered saline (PBS). The final pellet was suspended in 5 ml of PBS and sonicated using a Soniprep 150 (MSE) in 30 second pulses on ice. The lysate was decanted into microfuge tubes and centrifuged to clarify at 13000 rpm at 4^o^C and then the soluble lysate was aliquoted and stored at -20^o^C. The protein content was confirmed by SDS-PAGE and quantified using a Bradford assay compared to an albumin standard curve. The LPS content of the lysates was measured using the Limulus Amebocyte Lysate (LAL) kit (Thermo Scientific) and a modified in-house Mono Mac 6/Interleukin-6 *in vitro* pyrogen test (1).

**ELISA**

96-well ELISA plates (Nunc Maxisorp, Sigma-Aldrich, UK) were coated with selected antigen optimally diluted in PBS, and incubated overnight at 4^o^C. The plates were washed 3 times with PBS containing 0.05% Tween 20 (PBST) then blocked with 100 µl of 5% milk powder in PBST for 1 hour. After 3 washes, 100 μl of diluted sera in PBST containing 0.5% milk powder (diluent) was added and plates incubated for 2 h. After 6 washes, the plates were incubated for 1 h at RT with 50 μl of anti-human IgG/HRP (Dako polyclonal Rabbit P0214 Dako, Ely, Cambridgeshire) 1 in 5000 in diluent, and then washed 6 times, and developed with OPD (Sigma) substrate solution. The reaction was stopped with 25μl 2M H_2_SO_4_ and absorbance was read at 490 nm. Antigen-specific antibody isotypes were measured using isotype-specific biotinylated monoclonal antibodies followed by avidin-peroxidase (Sigma). Initial screening of sera at a 1in 25 dilution was carried out to establish the starting dilution of the sera based on the mean of absorbance obtained. A 4-step dilution series for each serum sample obtained an end-point titre. Inhibition ELISA was carried out to determine cross reactivity between antigens and hence specificity of the ELISA. Sera were pre-incubated with a dilution series of antigens before proceeding with the regular ELISA procedure. Measurement of Ig subclasses was performed by ELISA with the secondary detector antibody substituted to allow detection of the subclasses, including IgG1, IgG2, IgG3 and IgG4, as well as the other classes, IgA and IgM. In some instances, in which the secondary antibody was biotin-conjugated, an extra step was required, adding a dilution of Avidin–Peroxidase at 1in 40000 and incubating for 1h at RT prior to adding substrate. The absorbance (minus blank) value at a fixed serum concentration of 1in 25 was used as the result for the Ig subclasses.

**ELIspot**

Sterile 96-well ELIspot plates (Millipore, Watford.UK) were coated with 10 µg/ml of capture anti-human IFNγ (Mabtech, Nacka Strand, Sweden) in bicarbonate buffer overnight at 4^o^C. The plate was washed 3x with sterile PBS and blocked with 100 µl of RPMI 1640 supplemented with penicillin, streptomycin, glutamine and 10% foetal calf serum (R10) for 1h at RT. PBMC were thawed and counted using a CASY® cell counter (Roche Diagnostics GmbH, Mannheim, Germany), and adjusted to 8×10^6^/ml. Following decanting of the R10 block, 50 µl of stimulus and 50 µl of PBMCs were added to the plate. Stimuli comprised medium-only negative control, positive controls of anti-CD3 and phytohaemagglutinin (PHA), and bacterial antigens (Table S1). The plate was incubated for 20 h at 37°C in a 5% CO_2_ incubator. The plate was then washed 6 times before adding 50 µl of biotinylated-anti-IFNγ detector antibody at 1μg/ml (Mabtech). The plate was incubated for 2h at RT and was then washed, and 50μl of streptavidin-ALP (Mabtech) at 1μg/ml was added followed by 1 hour’s incubation. Finally, AP Conjugate Substrate kit 170-6432 (Bio-Rad Laboratories Inc), 50μl/well, was used to develop the plate for 10 minutes, and the reaction was stopped by washing the plate thoroughly with tap water. The plate was dried overnight and spot-forming cells were counted using an Immuno-Spot reader (Auto-immun Diagnostika GmbH, Strassberg, Germany). The frequency of IFNγ-producing cells was calculated from the number of spots in the wells after background subtraction and multiplied by the cell concentration factor (2.5 for 8x10^6^). The results are expressed as spot-forming cells (SFC)/10^6^(M) PBMC. To confirm that T cell responses were produced from T helper cells, experiments were carried out where CD4^+^ T cells were depleted before ELIspot using Dynal Beads, or anti-MHC antibodies were incubated with PBMC prior to and during the ELIspot incubation.

**Cell activation and surface staining and Flow Cytometry**

Frozen PBMC samples were thawed, washed and resuspended in R10 and counted. 250 μl of 8×10^6^ PBMCs were incubated with 250 μl of medium, polyclonal anti-CD3mAb (Mabtech) as positive control, or lysates + peptides from *P.aeruginosa* or *H.influenza*e. The cultures were incubated for 20h at 37°C, 5% CO_2_. The cells were washed with PBS and incubated (15 min, at RT, in the dark) with the antibody combinations shown in Table S1, together with 50 μl of Brilliant Stain Buffer (BD Horizon™). The stained cells were washed with 2 ml of PBS and re-suspended in 300μl of PBS prior to immediate acquisition on the flow cytometer. A BD FACSCanto^TM^ II flow cytometer (BD Biosciences, Oxford, UK) with 8 channels was used with BD FACSDiva^TM^ software.

**Staining for intracellular (IC) molecules**

Intracellular staining was performed to determine the number and phenotype of IFNγ-producing or activated CD69^+^ T cells following stimulation. T cells were activated as above for 20h, the last 18 h of which was in the presence of 0.1 µg/ml Brefeldin A (Golgi plug, BD Bioscience, Oxford, UK) to allow the cytokines to accumulate inside the cells. The cells were then first stained for surface molecules (Table S1) for 20 min, prior to being fixed with 100μl of IC Fixation Buffer (Affymetrix eBioscience, Hatfield, UK). Cells were incubated for 20 min in the dark at RT. Cells were then washed twice with 2ml of Permeabilization Buffer (Affymetrix eBioscience) and re-suspended in 100μl Permeabilization Buffer. 2 μl of IFNγ-PE (Affymetrix eBioscience) was added to the cells and followed by incubation for 30 min in the dark at RT. The cells were washed twice: once in Permeabilization Buffer and once in PBS. The cells were then re-suspended in 300μl PBS and acquired immediately on the flow cytometer.

**Multiplex cytokine assay**

For each panel, 1 ml of diluent 1 was added to 1 g of blocker B. Serial dilutions of 1in100 were made for each standard for both panels. Then, 25 μl of these standards were added to the appropriate 96-well plate (Meso Scale Diagnostic, LLC, Gaithersburg, USA). 25 μl of culture supernatant samples from previously stimulated PBMCs were added to each well of the 96-well plates. The plates were covered with an adhesive cover (Thermo Scientific, UK) and incubated for 2h at RT on a plate shaker (100 rpm). 25 μl of detector antibodies were added in pro-inflammatory panel 1 (human calibrator blend) including anti-human IL-1β, IL-2, IL-4, IL-6, IL-8, IL-10, IL-12p70, TNFα. 25 μl of detector antibodies was also added to cytokine panel 2 (human calibrator blend): anti-human IL-5 and IL-17A. These detection antibodies (Sulfo-TAG) were diluted in diluent 100 (MSD). The plates were incubated for 2h at RT on a plate shaker (100 rpm). The plates were washed 6 times with 150 μl of PBS 0.05% Tween-20. Then, 150 μl of read buffer 4X with surfactant (MSD) diluted in sterilized water (50:50) was added to each well of the plates. The plates were read on MSD sector imager. Data were analysed in MSD Software and Excel. Cytokine detection levels were 0 – 10ng/ml.

**References**

1.Poole S, Mistry Y, Ball C, Gaines Das RE, Opie LP, Tucker G, Patel M. A rapid 'one-plate' in vitro test for pyrogens. J Immunol Methods. 2003;274:209-20.

Table S.1. Microbial species used for antigens in the antibody and T cell assays

| Abbreviation code | Species | Gram | Strain ID and/or source | Growth conditions (agar plates/medium) |
| --- | --- | --- | --- | --- |
| MCAT | *Moraxella catarrhalis* |  | Wild9 (in house) | BHI, 37 ⁰C, 200 RPM |
| PSA | *Pseudomonas aeruginosa* | Neg | NCTC10662 | SCFM, 37 ⁰C, 200 RPM |
| SPN | *Streptococcus pneumoniae* | Pos | DMSZ11865 | BHIS, 37 ⁰C, 200 RPM |
| (NT) Hi | Non-typeable *Haemophilus influenzae* | Neg | MQCL 491 | Chocolate blood agar, 37, carbon dioxide on solid media or meat broth, 37, carbon dioxide on liquid media |
| HBO | *Haemophilus influenzae* serotype type B | Neg | (HbO-HA antigen)  NIBSC, Potters Bar,UK | n/a |

Note: Strains were either obtained from the National Collection of Typed Cultures (NCTC), Laboratory of Microbiology, University of Ghent (LMG) Belgium, or were fully identified wild in-house isolated and characterized strains.

**Table S.2** Fluorochrome labelled antibodies used in this study

| Fluorochromes | Cell markers | Function of  Molecule | Clone | Laser (nm) Excitation | EM-max^a^ (nm) (detection) |
| --- | --- | --- | --- | --- | --- |
| FITC | OX40^b^ | Co-stimulation | ACT35 | 488 | 525 |
| PE | CD49d^b^ | Homing | 9F10 | 488 | 575 |
| PerCP-Cy5.5 | CXCR3^c^ | Chemokine receptor (Th1) | IC6/CXCR3 | 488 | 695 |
| PE-Cy7 | CCR6^b^ | Chemokine receptor (Th17) | B6H | 488 | 785 |
| APC | PD-1^b^ | Senescence | eBioJ105 | 633 | 660 |
| APC-Cy7 | CCR5^c^ | Chemokine receptor (inflammatory) | 2D7/CCR5 | 633 | 785 |
| BV421 | CD69^c^ | Activation marker | KN50 | 405 | 421 |
| BV510 | CD4^c^ | Th cell marker | SK3 | 405 | 510 |

Notes: ^a^ Maximum emission wavelength for detection; ^b^ eBioscience (Affymetrix - eBioscience, Hatfield, UK); ^c^ BD Biosciences (Oxford, UK).


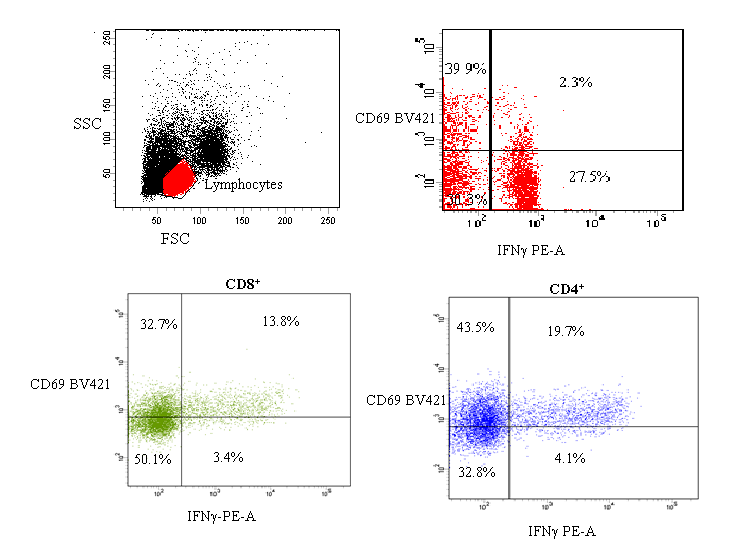


CD8

CD4

a

b

c

d

**Figure S1. Gating of activated CD8^+^ and CD4^+^ T cells**

Forward scatter vs side scatter (a) allowed lymphocytes to be gated upon. Lymphocytes were then gated upon based on their expression of CD8 or CD4 (b). CD8^+^ cells with intracellular staining for CD69 and IFNγ (c). CD4^+^ cells with intracellular staining for CD69 and IFNγ (d). All quadrants were based on the matched-fluorochrome isotype controls.
